# Antiatherosclerotic Potential of Active Principle Isolated from *Eugenia jambolana* in Streptozotocin-Induced Diabetic Rats

**DOI:** 10.1155/2011/127641

**Published:** 2011-04-07

**Authors:** Reenu Singh Tanwar, Suman Bala Sharma, Usha Rani Singh, Krishna Madhava Prabhu

**Affiliations:** ^1^Department of Biochemistry, University College of Medical Sciences, University of Delhi, Delhi 110095, India; ^2^Department of Pathology, University College of Medical Sciences, University of Delhi, Delhi 110095, India

## Abstract

The aim of the present study was to investigate the antiatherosclerotic effect of active principle (FIIc) isolated from aqueous fruit pulp extract of *Eugenia jambolana*. Crude aqueous extract of *E. jambolana* was subjected to purification using chromatographic techniques which yielded purified active compound (FIIc). Purity of FIIc was tested by HPLC. Phytochemical investigation of FIIc by NMR, IR, and UV spectra showed that the purified compound is *α*-*hydroxy succinamic acid*. The streptozotocin- (STZ-) induced diabetic rats were fed atherosclerotic (Ath) diet containing 1.5 mL olive oil containing 8 mg (3, 20,000 IU) vitamin D_2_ and 40 mg cholesterol for 5 consecutive days. The STZ-induced diabetic rats receiving Ath diet were orally administered FIIc at doses of 10, 15, and 20 mg/kg, and results were compared with reference drug, that is, glibenclamide (600 *μ*g/mg) and healthy control. 30-day treatment with FIIc resulted in significant (*P* < .001) improvement in blood glucose, serum lipid profile, apolipoproteins (Apo A_1_ and apoB_100_), and endothelial dysfunction parameters. Histomorphological studies also confirmed biochemical findings. Our results showed that FIIc has protective effect on hyperglycemia-induced atherosclerosis.

## 1. Introduction

Out of a large number of herbal drugs which possess antidiabetic activity in the Ayurvedic system of medicine of India, *Eugenia jambolana* is being widely used to treat diabetes by the traditional practitioners over many centuries [1]. It belongs to family Myrtaceae, (called Black plum/Black berry in English and Jamun in Hindi in India).

The antihyperglycemic activity of seeds of *E. jambolana* is well documented [2–11]. Studies on antihyperglycemic activity of the fruit pulp of *E. jambolana* are not much reported. Achrekar et al., 1991 [4] first claimed that the water extract of fruit pulp of *E. jambolana* showed hypoglycemic activity immediately or as early as 30 minutes, while seeds required 24 hours for the same effect. Hot water extract of dried fruit pulp was found to be inactive in alloxan-induced hyperglycemia [2]. In our previous studies [12], water extract of *E. jambolana* was found to be more potent in reducing the fasting blood glucose in comparison to the ethanolic fruit pulp extract. However scientific studies are lacking regarding antiatherosclerotic activity of active compound isolated from fruit pulp of *E. jambolana*. Survey of literature showed that traditional medicinal plants having antidiabetic properties can be used as drugs or simple dietary adjuvant to existing therapies of diabetes and its associated complications like atherosclerosis [13–19]. In our lab, active compound (FIIc) has already been isolated (US Patent no. 6,426,826 dated 6th August 2002; Indian Process Patent no. 1,88,759 May 2003; Indian Product Patent no. 2,30,753 February 2009). Hence an attempt has been made to investigate the effect of purified active principle (FIIc) on hyperglycemia-induced atherosclerosis.

## 2. Materials and Methods

### 2.1. Plant Material

 Fruits of *E. jambolana* were procured from the Azadpur Mandi (Herbal market) at Delhi. The identity was made with the help of a botanist using taxonomic rules (voucher specimen no. P-96/6) and specimen is kept for further references in Botanical Garden, Kolkata, India.

### 2.2. Preparation of Crude Aqueous Extract

Fresh fruits of *E. jambolana* were purchased from local market and washed thoroughly. Seeds were separated from fruit pulp of *E. jambolana*. The pulp was ground for 10 minutes in a mixer with distilled water (500 mL). It was allowed to stand overnight at 4°C. The pulp was then filtered through 5-6 layers of muslin cloth. The whole procedure was carried out in cold room at 4°C. The filtrate was first centrifuged for 15 minutes in a refrigerated centrifuge at 10,000 rpm at 4°C and then lyophilized to store it for a longer duration. The yield of lyophilized water extract was about 10 g from 650 g of fruit pulp, which was obtained from 1 kg fruits of *E. jambolana*.

### 2.3. Isolation and Purification of Active Antihyperglycemic Compound FIIc

The lyophilized aqueous extract of pulp was subjected to isolation and purification of antihyperglycemic compound. The purification was done by ion exchange column chromatography using diethylaminoethylcellulose-52 (DEAE-52) as the column material. Fractions were then eluted with 0.1 M phosphate buffer (pH 6.0). The first colourless fraction (FI) showed hyperglycemic activity. Then after elution of some inactive material, an antihyperglycemic fraction (FII) was obtained. This was followed by fractions FIII and FIV. The separation of FII and FIII was clear-cut without overlapping. As FII was found to have potent antihyperglycemic activity. It was further subjected to purification by rechromatography and active compound FIIc was obtained (as discussed in the introduction patents have already been granted for the isolation of active principle).

### 2.4. Chemical Characterization of Active Antihyperglycemic Compound FIIc

**Figure 1:**
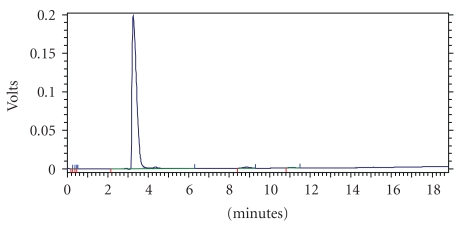
HPLC fingerprint of active principle isolated from fruit pulp of *Eugenia jambolana. *

Homogeneity of FIIc was confirmed by HPLC which gave a single peak after employing it on chromolith column (chromolith performance HPLC column RP-18e 100–4.6 mm). FIIc was eluted with mobile phase (water : methanol : Acetonitrile:: 70 : 15 : 15) and monitored by PDA detector at wavelength 220 nm (Instrument Shimadzu HPLC model SPD-M20A). A single peak was observed in chromatogram, suggesting that FIIc was almost homogenous (Figure 1). 

Purified antihyperglycemic compound FIIc is soluble in water and on heating, burns with a nonsmoky flame. Elemental analysis of purified hypoglycemic compound showed the presence of carbon, hydrogen, oxygen, and nitrogen with a ratio of carbon to nitrogen of 4 : 1, respectively. One-dimensional ^I^H NMR spectra showed four signals at *δ* 2.4(dd), 2.66(dd), 3.66(S), and at *δ* 4.38 (dd). Type ^13^C NMR showed fine signals of *δ* 45.5, 65.3, 73.0, 182.7, and 184.0. The signals at 182.7 and 184 were described to be carbonyl containing carbon. IR spectrum showed the presence of bands at 1656.0 and 1590.2 which confirmed the presence of amide and acid moiety, respectively. 

Detailed UV, NMR, and IR spectrum suggested that the purified active compound (FIIc) is **α*-hydroxy succinamic acid*, which is a small aliphatic organic compound having molecular formula C_4_H_7_O_4_N.

### 2.5. Experimental Animals

Male Wistar albino rats (weighing 160–200 g) were procured from Central Animal House of University College of Medical Sciences (UCMS), University of Delhi, Delhi, India. The animals were housed in standard conditions of temperature (22 ± 2°C) and at 12 h light-dark cycle. The rats were fed with commercial diet (Hindustan liver Ltd., Mumbai) and water *ad libitum*. The experimental protocol was approved by the Institutional Animal Ethical Committee (IAEC) of UCMS, Delhi, India. All experimental procedures were conducted in accordance with the ethical guidelines of International Association for the Study of Pain [20].

### 2.6. Experimental Induction of Diabetes

 A freshly prepared solution of streptozotocin (45 mg/kg in 0.1 M citrate buffer, pH 4.5) was injected intraperitoneally to overnight fasted rats. Streptozotocin- (STZ-) injected animals exhibited hyperglycemia within 48 hrs [21]. Fasting blood glucose (FBG) levels were measured after 48 hours and again repeated twice at an interval of three days. The rats with stabilized diabetes having FBG values of 250 mg/dL or above were considered diabetic and were included in the study.

### 2.7. Sample Collection

 Blood was drawn from retro-orbital plexus of overnight fasted rats by using microcapillary technique [22]. Blood samples were collected in anticoagulant (sodium fluoride and potassium oxalate) vials for plasma separation to estimate blood glucose. For estimation of soluble vascular cell adhesion molecule-1 (sVCAM-1) and fibrinogen, whole blood was collected in EDTA vial and separated plasma was used. The blood collected in plain vials, was allowed to clot for separation of serum. Serum was used for estimation of serum lipid profile parameters, oxidized LDL (Ox-LDL), apolipoproteinA_1_ (ApoA_1_) and apolipoproteinB_100_ (ApoB_100_), and serum total NO levels. Heart and aorta were excised and preserved in 10% neutral formalin for histomorphological studies.

### 2.8. Analytical Methods

Blood glucose was measured using the glucose oxidase-peroxidase method [23]. The levels of sVCAM and fibrinogen were estimated by Enzyme Linked Immuno Sorbant Assay (ELISA) using commercially available kits from Diaclone France and Hyphen Biomed, France, respectively. Triglycerides (TG) were measured by the method of Fossati and Prencipe, 1982 [24]. Total cholesterol (TC) was assayed as per the method of Allain et al., 1974 [25]. High-density lipoprotein cholesterol (HDL-C) was determined by Burstein et al.'s method [26]. Low-density lipoprotein cholesterol (LDL-C) and very low-density lipoprotein cholesterol (VLDL-C) were calculated by using the formula of Friedwald et al. [27]. ApoA_1_ and ApoB_100_ were estimated by immunoturbidimetry method using commercially available kits from Giesse diagnostics. NO end products and Ox-LDL were estimated as per the method of Moshage et al., 1995 [28] and Ahotupa et al., 1996 [29].

### 2.9. Experimental Design

The experiment was carried out on following groups of seven rats in each group. Atherosclerotic (Ath) diet includes 1.5 mL olive oil containing 8 mg (3,20,000 IU) vitamin D_2_ and 40 mg cholesterol and was given for 5 consecutive days. Group 1: healthy control; Group 2: diabetic control + Ath diet; Group 3: diabetic + Ath diet + Glibenclamide (600 *μ*g/kg); Group 4: diabetic + Ath diet + FIIc (10 mg/kg); Group 5: diabetic + Ath diet + FIIc (15 mg/kg); Group 6: diabetic + Ath diet + FIIc (20 mg/kg).

Control rats (groups 1 & 2) received vehicle, that is, distilled water. Group 3 received the glibenclamide and groups 4, 5, and 6 received FIIc at the above-mentioned dose dissolved in 1 mL of distilled water. The treatment was given daily for a period of 30 days using standard orogastric cannula. Fasting blood glucose levels were measured at 0, 6, 10, 20, and 30 days. All other parameters were determined at day 0, day 6, and day 30.

### 2.10. Histomorphological Studies

For histomorphological studies, heart and aorta were dissected out and washed thoroughly with physiological saline to remove adherent fats and blood. Both the tissues were grossly examined. The sections of heart and aorta were taken for paraffin blocks. Sections were cut at 4-5 microns and stained with routine haematoxylin and eosin stains. These slides were evaluated under light microscope for histomorphological examination.

### 2.11. Statistical Analysis

Values are expressed as the mean ± SEM for seven animals in each group. The data was analyzed by using repeated measure analysis of variance (ANOVA) followed by Dunnett's test and repeated measure ANOVA followed by Tukey's test. The results were considered significant at *P* < .05.

## 3. Results

### 3.1. Glycemic Control

There was dose-dependent fall in the levels of FBG in diabetic rats treated with FIIc (10, 15, and 20 mg/kg) from day 6 to day 30. Untreated diabetic rats showed marked hyperglycemia throughout the experimental period (Table 1). As the FIIc produced dose-dependant fall in blood glucose level up to 15 mg/kg, this dose was considered as effective dose and further studies were carried out with 15 mg/kg dose of the FIIc. The diabetic rats fed with the FIIc and glibenclamide exhibited remarkable glycemic control as evident by significant decrease (*P* < .001) in FBG.

**Figure 2:**
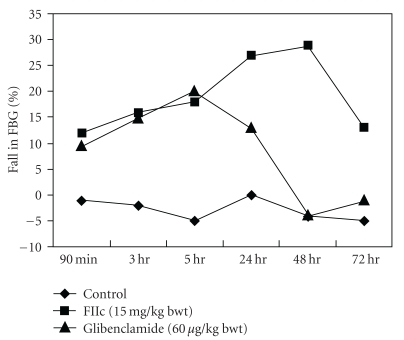
Effect of single dose of FIIc (15 mg/kg) on fasting blood glucose at different time interval in diabetic rats. Effect of glibenclamide (600 *μ*g/kg) is shown for comparison. The rats were treated with a single dose of drugs, and FBG were measured at different time points, that is, from 90 min to 72 hr. In FIIc treated group, FBG level gradually declined up to 48 hr, whereas in glibenclamide-treated group, gradual fall in FBG level was observed only up to 5 hr.

As shown in Figure 2, the oral administration of single dose of FIIc (15 mg/kg) exhibited a gradual decline in FBG level from 90 min to 48 hr and thereafter rise in FBG was observed. The antihyperglycemic effect of FIIc (15 mg/kg) was better than that of glibenclamide as shown in Figure 2.

### 3.2. Lipidemic Control

**Figure 3:**
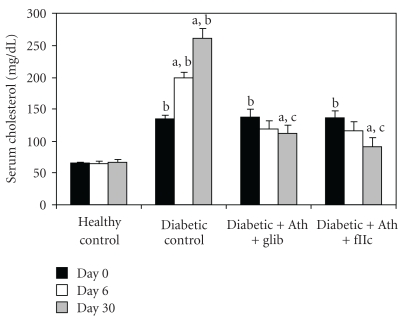
Effect of oral administration of FIIc (15 mg/kg) on serum cholesterol after 30-day treatment. Data are expressed as mean ± SEM of seven animals in each group. ^a^ *P* < .001 versus initial values; ^b^ *P* < .001 versus normal control; ^c^ *P* < .001 versus diabetic control.

**Figure 4:**
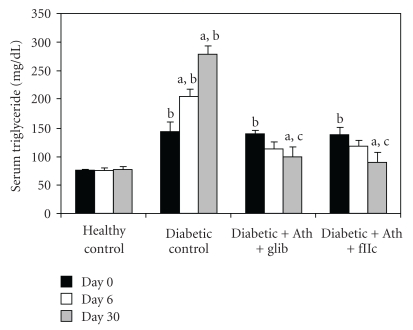
Effect of oral administration of FIIc (15 mg/kg) on serum triglyceride after 30-day treatment. Data are expressed as mean ± SEM of seven animals in each group. ^a^ *P* < .001 versus initial values; ^b^ *P* < .001 versus normal control; ^c^ *P* < .001 versus diabetic control.

**Figure 5:**
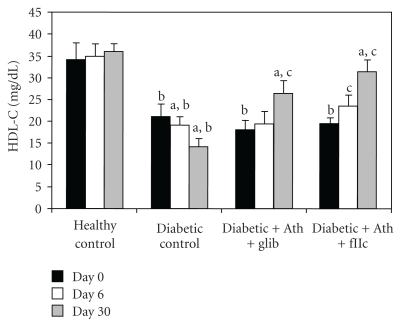
Effect of oral administration of FIIc (15 mg/kg) on HDL-C after 30-day treatment. Data are expressed as mean ± SEM of seven animals in each group. ^a^ *P* < .001 versus initial values; ^b^ *P* < .001 versus normal control; ^c^ *P* < .001 versus diabetic control.

**Figure 6:**
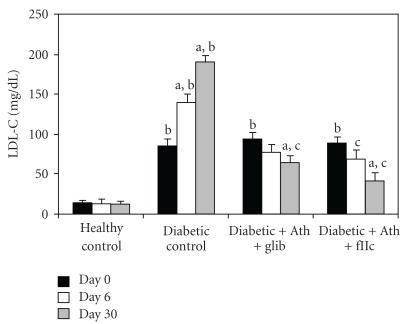
Effect of oral administration of FIIc (15 mg/kg) on LDL-C after 30-day treatment. Data are expressed as mean ± SEM of seven animals in each group. ^a^ *P* < .001 versus initial values; ^b^ *P* < .001 versus normal control; ^c^ *P* < .001 versus diabetic control.

**Figure 7:**
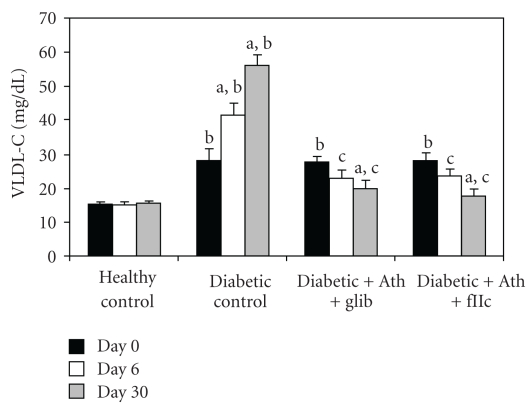
Effect of oral administration of FIIc (15 mg/kg) on VLDL-C after 30-day treatment. Data are expressed as mean ± SEM of seven animals in each group. ^a^ *P* < .001 versus initial values; ^b^ *P* < .001 versus normal control; ^c^ *P* < .001 versus diabetic control.

Diabetic control animals showed significant increment (*P* < .001) in the levels of TC (Figure 3), TG (Figure 4), LDL-C (Figure 6) & VLDL-C (Figure 7) and reduction (*P* < .001) in HDL-C (Figure 5) compared with healthy control. Treatment with FIIc for 30 days significantly reduced (*P* < .001) the levels of TG, TC, LDL-C, and VLDL-C. The significant (*P* < .001) improvement was also observed in HDL-C after treatment with FIIc. Moreover there was significant improvement in the levels of ApoA_1_, ApoB_100_, and ApoB_100_/ApoA_1_ ratio (Table 2). The effect produced by FIIc was better than that of glibenclamide.

### 3.3. Endothelial Dysfunction Parameters

**Figure 8:**
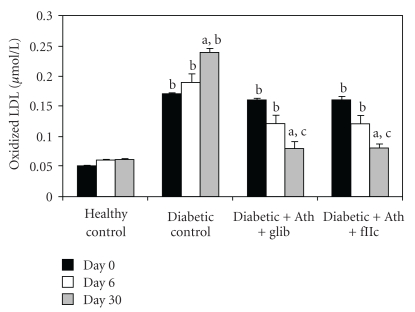
Effect of oral administration of FIIc (15 mg/kg) on Ox-LDL after 30-day treatment. Data are expressed as mean ± SEM of seven animals in each group. ^a^ *P* < .001 versus initial values; ^b^ *P* < .001 versus normal control; ^c^ *P* < .001 versus diabetic control.

**Figure 9:**
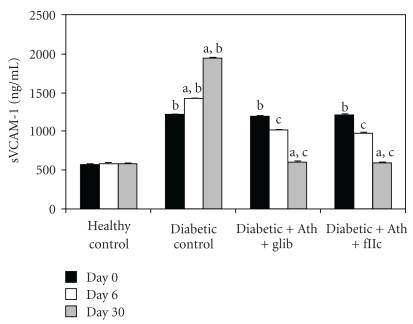
Effect of oral administration of FIIc (15 mg/kg) on sVCAM-1 after 30-day treatment. Data are expressed as mean ± SEM of seven animals in each group. ^a^ *P* < .001 versus initial values; ^b^ *P* < .001 versus normal control; ^c^ *P* < .001 versus diabetic control.

**Figure 10:**
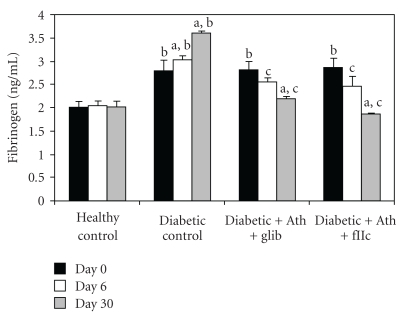
Effect of oral administration of FIIc (15 mg/kg) on Fibrinogen levels after 30-day treatment. Data are expressed as mean ± SEM of seven animals in each group. ^a^ *P* < .001 versus initial values; ^b^ *P* < .001 versus normal control; ^c^ *P* < .001 versus diabetic control.

A significant elevation (*P* < .001) in the levels of Ox-LDL (Figure 8), sVCAM-1 (Figure 9), and fibrinogen (Figure 10) was observed in diabetic control rats as compared to normal rats. The Ox-LDL was also found to be significantly decreased (*P* < .001) in FIIc-fed diabetic rats. The supplementation with the FIIc reverted back the sVCAM-1 and fibrinogen levels to near normal in diabetic rats, which was statistically significant at *P* < .001. Glibenclamide-treated diabetic rats also produced significant reduction (*P* < .001) in the levels of oxidized LDL, sVCAM-1, and fibrinogen.

**Figure 11:**
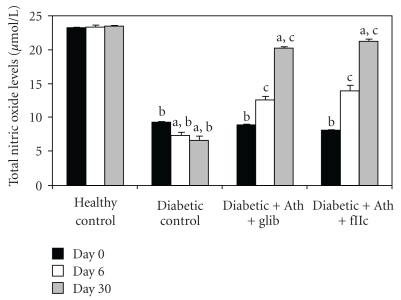
Effect of oral administration of FIIc (15 mg/kg) on Total No levels after 30-day treatment. Data are expressed as mean ± SEM of seven animals in each group. ^a^ *P* < .001 versus initial values; ^b^ *P* < .001 versus normal control; ^c^ *P* < .001 versus diabetic control.

As shown in Figure 11, the total nitric oxide levels in diabetic control animals was found to be significantly lower (*P* < .001) than in healthy control group. The protective effect of FIIc is indicated by the significant increase (*P* < .001) in levels of total nitric oxide of FIIc-fed diabetic rats. The effect of glibenclamide on endothelial dysfunction parameters was noticed at *P* < .001 for nitric oxide levels, serum nitrite, and serum nitrate in diabetic rats. Diabetic control animals showed significantly (*P* < .001) lowered nitric oxide levels when compared with healthy control.

### 3.4. Histomorphological Studies

**Figure 12:**
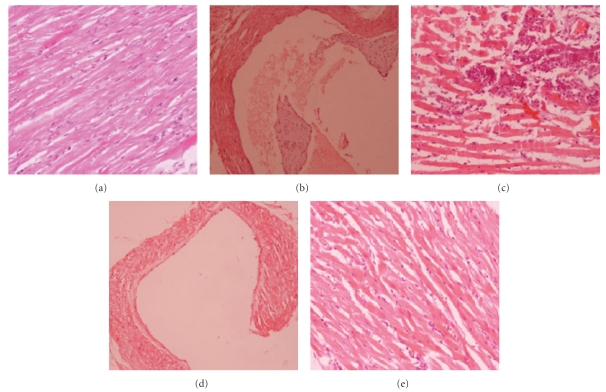
(a) Histomorphological study of healthy rats heart showing normal morphology (haematoxylin and eosin (H-E) stain, original magnification 20x). (b) Diabetic control rats (H-E stain, original magnification 20x) showing atherosclerotic plaque. (c) Diabetic control rats (H-E stain, original magnification 20x) showing large areas of inflammation. (d) Diabetic rats treated with active principle FIIc isolated from *Eugenia jambolana* fruit pulp (H-E stain, original magnification 20x) showing morphology of heart chamber within normal limits. (e) Diabetic rats treated with FIIc (H-E stain, original magnification 20x) showing lesser inflammation.

Histomorphological examination of normal healthy control group showed heart and aorta within normal limits (Figure 12(a)). Histomorphological examination of tissue from diabetic rats showed atherosclerotic lesions and large areas of vacuolization and inflammation that are both acute and chronic inflammations (Figures 12(b) and 12(c)). The inflammation was predominantly by mononuclear cells comprising of lymphocytes, histocytes, and plasma cells.

After 30-day treatment with FIIc, inflammation in myocardium was reduced as compared to diabetic control. After 30-day treatment with FIIc, normal histology of heart was observed in FIIc treated animals (Figures 12(d) and 12(e)).

## 4. Discussion

Atherogenicity with subsequent cardiovascular manifestations is one of the important causes of high mortality and morbidity. Various agents which affect hyperlipidemia are still not used for prevention of atherosclerosis, because of their potential toxicity and intolerance [30]. Antihyperglycemic effect of aqueous extract of *E. jambolana* fruit pulp in normal and alloxan-induced (80 mg/kg) diabetic rabbits have already been shown in our previous study [12]. The main goal of the present study was to purify active principle from aqueous fruit pulp extract and find its efficacy against diet-induced atherosclerosis in diabetic rats.

In the present study, we observed that the antihyperglycemic effect of FIIc starts to come from 90 min and gradually increases (Figure 2). In separate groups of animals, it was found that fall in FBG steadily occurs up to 48 hr with a single dose of 15 mg/kg, and thereafter increases in diabetic rats. This also showed that the antihyperglycemic effect of FIIc persists till 48 hr with its single dose (15 mg/kg), which was not observed in glibenclamide-treated rats. After 30-day treatment, the FIIc like glibenclamide produced significant reduction in blood glucose level of STZ-induced diabetic rats. Capacity of FIIc to significantly bringing down the elevated levels of blood glucose in diabetic rats shows its antihyperglycemic activity, which is an essential trigger for the development of normal homeostasis during experimental diabetes and its associated complications. Since hyperlipidemia is one of the major risk factors for the development of atherosclerosis. The increased TG and TC levels and decreased HDL-C are known risk factors for coronary heart disease (CHD) [31, 32]. Lipids and apoprotein moieties of LDL particle may be damaged in reaction with free radicals (oxidized LDL). Increased postsynthetic chemical modification of LDL by oxidation, glycosylation, or both may induce endothelial injury and/or accelerate foam cell formation by monocyte macrophage in arterial intima [33]. Plasma ApoB_100_ concentration reflects the number of atherogenic lipoproteins and studies done so far demonstrated that ApoB_100_ can be a valuable predictor for coronary artery disease (CAD) [34, 35]. Recent studies have shown that the ApoB_100_/ApoA_1_ ratio is strongly associated with risk of CAD [36]. The altered lipid and lipoprotein profile, that is, increase in TG, TC and LDL-C with fall in HDL-C was reversed towards normal level along with significant improvement in Ox-LDL, ApoA_1_, ApoB_100_ and ApoB_100_/ApoA_1_ ratio after administration of the FIIc for 30 days. As these factors showed significant improvement following treatment with the FIIc, it may be suggested that the FIIc may help to prevent the progression of cardiovascular diseases. 

Endothelial dysfunction with its proinflammatory and prothrombotic phenotype is a key element in the pathogenesis of atherosclerosis in humans [37]. Abnormalities of NO/O_2_ pathway occur in diabetes and are important cause of endothelial dysfunction. Serum total NO levels (nitrite + nitrate) were found to be decreased in type 2 diabetic subjects [38]. In the present study, which is in accordance with various previous studies, serum total NO levels were significantly (*P* < .001) decreased in diabetic control rats and treatment with FIIc significantly (*P* < .001) improved the total NO levels. VCAM-1 is a transmembrane glycoprotein, which is a member of the immunoglobulin gene superfamily. These molecules play an important role in adhesion of circulating leukocytes to endothelium, which is the first step in initiation of atherosclerosis [38]. The pathogenic mechanism (s) whereby fibrinogen acts as a cardiovascular risk factor is likely to be multifactorial. Plasma fibrinogen levels are associated with platelet aggregability [39] which is important in the genesis of a vascular lesion [40]. In the present study, circulating levels of sVCAM-1 and fibrinogen were significantly high in diabetic control rats compared to healthy control (*P* < .001) and treatment with FIIc significantly improved the circulating levels of sVCAM-1 as well as fibrinogen.

The antihyperglycemic and dyslipidemic effect of FIIc was comparable to that of glibenclamide. However, the effect of the FIIc on endothelial dysfunction parameters was found to be better than that of glibenclamide. In conclusion, the FIIc exhibited potential antihyperglycemic and antiatherosclerotic activity in diabetic animals. The results of the present study provides impetus for further molecular and mechanistic studies on the therapeutic action of FIIc, before it can be administered as possible adjuvant therapy for reducing atherosclerotic complications in diabetes.

## Figures and Tables

**Figure 13:**
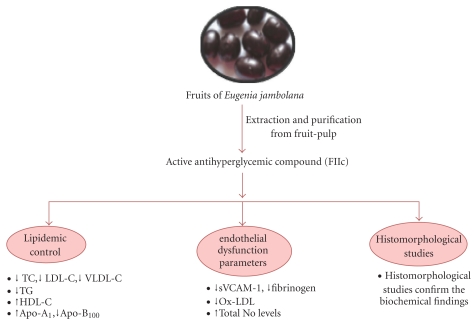
Therapeutic approach of FIIc purified from fruit pulp of *Eugenia jambolana *on experimental-induced atherosclerosis.

**Table 1:** Effect of oral administration of different doses of FIIc on fasting blood glucose (mg/dl) at various time intervals

Group	Dose	Day 0	Day 6	Day 10	Day 20	Day 30
Healthy control	—	83.10 ± 4.5	79.50 ± 10.2	84.90 ± 5.0	83.70 ± 7.6	83.50 ± 8.0
Diabetic control	—	301.5 ± 17.1	306.3 ± 12.9	313.7 ± 13.9^a^	322.4 ± 10.3^a^	326.3 ± 9.8^a^
Diabetic + Glibenclamide	600 *μ*g	302.9 ± 16.4	259.5 ± 39.5^a^	208.3 ± 21.9^a^	187.1 ± 23.2^a^	144.1 ± 17.9^a^
Diabetic + FIIc	10 mg	302.6 ± 13.1	300.9 ± 19.6	276.0 ± 25.4^a^	213.3 ± 28.3^a^	187.8 ± 16.8^a^
Diabetic + FIIc	15 mg	306.5 ± 14.9	269.0 ± 31.2^a^	228.5 ± 26.7^a^	155.1 ± 27.4^a^	121.2 ± 16.8^a^
Diabetic + FIIc	20 mg	301.8 ± 12.1	298.1 ± 27.3	259.8 ± 26.2^a^	195.4 ± 30.2^a^	132.3 ± 18.6^a^

Data are expressed as mean ± SEM of five animals in each group and evaluated by repeated measure ANOVA followed by Dunnett's test.

^a^ *P* < .001 versus day 0.

**Table 2:** % Change in Apolipoprotein-A_1_, Apolipoprotein-B_100_, and Apolipoprotein B_100_/Apolipoprotein-A_1_ ratio after 30 days.

Parameters	Normal control	Diabetic control	Diabetic + Ath diet + Glibenclamide	Diabetic + Ath diet + FIIc
Apolipoprotein-A_1_	—	42% ↓	9.3% ↑	10.4% ↑
Apolipoprotein-B_100_	—	10.2% ↑	16.9% ↓	21.5% ↓
Apo-B_100_/A_1_ ratio	—	49.1% ↑	24.8% ↓	27.8% ↓
